# The Emerging Role of Artificial Intelligence and Automated Platforms for the Assessment of Penile Curvature: A Scoping Review

**DOI:** 10.1007/s11934-024-01232-6

**Published:** 2024-09-20

**Authors:** Kieran Lewis, Lydia DeAngelo, Omer Raheem, Raevti Bole

**Affiliations:** 1 Glickman Urological and Kidney Institute, Cleveland Clinic, Cleveland, OH USA; 2 Cleveland Clinic Lerner College of Medicine, Cleveland Clinic, Cleveland, OH USA; 3 Department of Urology, The University of Chicago Medical Center, Pritzker School of Medicine, Chicago, IL USA; 4 Glickman Urological and Kidney Institute, Cleveland Clinic, Cleveland, OH USA

**Keywords:** Peyronie’s Disease, Penile Curvature, Penile Angle Estimation, Artificial Intelligence, Hypospadias Repair

## Abstract

**Purpose of the Review:**

The estimation of penile curvature is an essential component in the assessment of both Peyronie’s disease and hypospadias-associated congenital penile curvature, as the degree of curvature can significantly impact treatment decision-making. However, there is a lack of standardization in curvature assessment and current methodologies are prone to inaccuracies. With the rise of artificial intelligence (AI) in urology, new research has explored its applications in penile curvature assessment. This review aims to evaluate the current uses of AI and other automated platforms for assessing penile curvature.

**Recent Findings:**

Several novel and promising tools have been developed to estimate penile curvature, some utilizing AI-driven models and others employing automated computational models. These platforms aim to improve curvature assessment in various settings, including at-home evaluation of Peyronie’s disease, in-office assessments using three-dimensional (3D) methodologies, and preoperative evaluations for hypospadias repair. In general, these new platforms produce highly accurate and reproducible angle estimates in non-clinical studies, however their effectiveness and relation to patient outcomes has had limited evaluation in clinical settings.

**Summary:**

Significant advancements have been made in the assessment and estimation of penile curvature in both Peyronie’s and pediatric patients, largely driven by AI and other automated platforms. Continued research is needed to validate these findings in clinical studies, confirm their efficacy, and assess their feasibility for real-world applications.

**Supplementary Information:**

The online version contains supplementary material available at 10.1007/s11934-024-01232-6.

## Introduction

In recent years, the use of artificial intelligence (AI) has exploded in the field of urology, with current clinical applications in radiomics, cancer detection, outcomes prediction, and more [1]. This change has been particularly apparent in the management of penile curvature associated with Peyronie’s disease and congenital penile curvature. The accurate and objective estimation of curvature angle, an important aspect of clinical decision-making, has significantly benefited from advancements in image recognition and analysis, largely driven by deep learning models [2]. As these AI-driven technologies continue to advance, their potential to improve clinical care in conditions like Peyronie’s disease becomes increasingly evident.

Peyronie’s disease is an acquired and progressive curvature of the penis caused by fibrosis of the tunica albuginea [3]. The prevalence of the disease is varied, with estimates ranging from 0.3 to 8.9% in the general population and up to 20.3% in specific sub-populations [4, 5]. The condition is thought to arise through trauma or repeated micro-trauma to the penile shaft, although many individuals with the disease have no recollection of an inciting incident [6]. Peyronie’s disease can have significant physical and psychosocial impacts on patients, as the most common symptoms include pain with erections, erectile dysfunction, and difficulty engaging in intercourse due to the degree of curvature. The disease is typically split into the acute and chronic phase, with the acute phase characterized by active inflammation leading to progressive penile deformity and penile pain over a 6–18 month period. In contrast, the chronic phase is typically characterized by resolution of pain and stabilization of penile plaque, although the associated deformity persists. Surgical interventions for Peyronie’s are almost exclusively performed during the chronic phase.

The assessment of penile curvature is an essential component of disease monitoring, decision-making, and evaluation of therapeutic efficacy in patients with Peyronie’s disease [7]. Current data suggests that stability of curvature for at least three months is suggestive of chronic disease, and indicates possible candidacy for surgical intervention [8]. Additionally, current guidelines restrict recommended treatment options to specific degrees of curvature in those with stable disease. For example, American Urologic Association guidelines recommend intralesional collagenase to be considered for those with penile curvatures between 30° and 90° (3). European Association of Urology guidelines recommend incision and grafting procedures in situations where the degree of curvature is over 60° or in complex curvature. In contrast, Nesbit or plication procedures are considered for those with curvatures below 60° (8). As such, the accurate and objective estimation of angle curvature in Peyronie’s disease is an important component of clinical decision-making.

The gold standard for curvature assessment is often considered to be in-office intracavernosal injection (ICI) followed by measurement of the erect penis with a protractor or goniometer. However in practice, methods for angle estimation vary by practitioner and can include the use of a vacuum erection device for in-office estimation, subjective estimation by patients, or patient-submitted photographic assessment. Outside of in-office ICI, most methods are largely thought to be inaccurate and unreliable [9, 10]. ICI has its drawbacks as well, as it is relatively invasive, presents a risk for priapism, and can be uncomfortable for the patient.

Given these challenges, there is a clear need for novel, accurate, and reliable methods for the assessment of Peyronie’s disease, and with the rise of artificial intelligence in medicine, the field of penile curvature assessment has been rapidly growing. AI-based research has extended not only to Peyronie’s disease but also to congenital penile curvature, and in both fields, there have been promising developments in the objective and accurate estimation of penile curvature. This scoping review aims to summarize the current research on AI and other automated platforms for the assessment of penile curvature.

## Methods

### Search Strategy

A literature review was conducted utilizing the Preferred Reporting Items for Systemic Reviews and Meta-Analyses Extension for Scoping Reviews (PRISMA-ScR) methodology [11] (Supplementary Table 1). The search was conducted utilizing PubMed, Scopus, and Google Scholar databases. The following search was performed in March 2024 on PubMed databases: (“automated” or “artificial intelligence” or “machine learning” or “algorithm” or “neural networks” or “deep learning”) AND (“penile curvature” or “Peyronie” or “Peyronie’s disease” or “chordee” or “hypospadias”). Additional keyword searches were performed on Scopus and Google Scholar. The reference list of identified articles was also reviewed for additional relevant studies. No limits were placed on study years and only English language articles were reviewed.

### Selection Criteria

Studies were included if they reported primary data regarding the use of AI or other platforms for the automated estimation of penile curvature. Studies with both human subjects (e.g. Peyronie’s disease, congenital penile curvature) or non-human models were included. Study types considered for inclusion were randomized-control trials, observational, case-control, basic science, non-human studies, and abstracts. Review articles and unpublished data were not considered for inclusion.

### Screening and Data Collection

Articles identified in database searches were first screened based on their title and abstract. Based on abstract screening, articles considered for inclusion were downloaded and reviewed. After the selection of included articles, the following data items were extracted from the identified articles; objective, subjects, type of model or algorithm use, required inputs for model/algorithm, objective data regarding penile estimation, and comparison method. Data screening and abstraction were performed by a single author (KL) and confirmed by an independent author (RB).

## Results

### Search Results and Summary Statistics

**Fig. 1:**
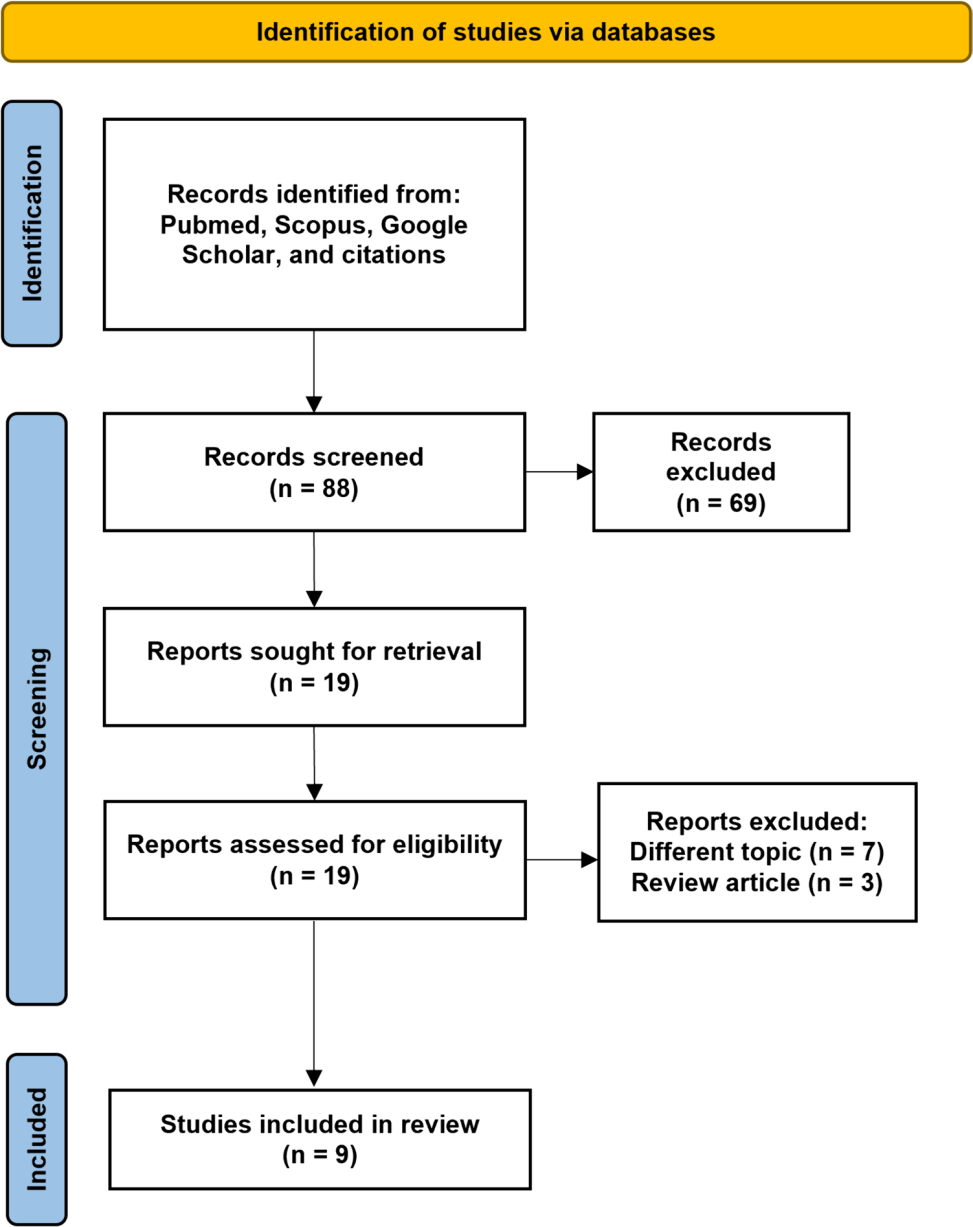
Screening and identification of studies eligible for inclusion

**Fig. 2:**
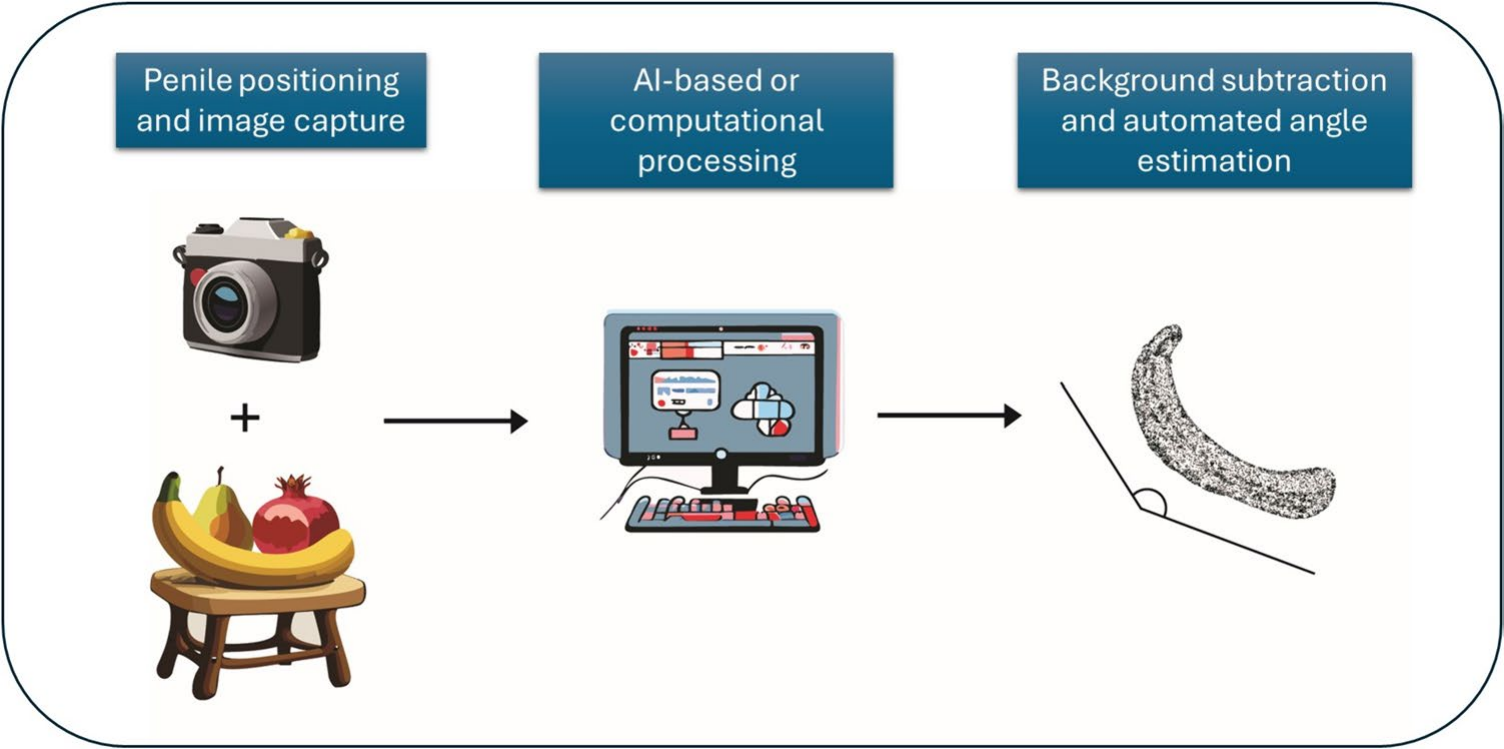
Workflow Illustration for AI-Based Penile Curvature Estimation. Illustrative depiction of the typical workflow for estimating penile curvature using AI-based or automated platforms. The process begins with image capture, employing either two-dimensional or 3D imaging. These images are then processed by AI or computational software, which refines the images through background subtraction and other techniques. Finally, the software outputs an automated estimation of the curvature angle

A literature search utilizing the above-described terms resulted in 88 articles. After initial screening of titles and abstracts, 19 studies were sought for retrieval and among these studies, 9 ultimately met the inclusion criteria (Fig. 1). Each study included had developed a platform for penile curvature estimation utilizing either AI or automated algorithms. The identified studies underwent a thematic analysis and were grouped according to the following; Automated at Home Curvature Assessment in Peyronie’s disease, 3D Imaging for Computational Assessment of Curvature, and Assessment of Congenital Penile Curvature. Of the studies identified, four were focused on building tools for the Peyronie’s disease population and five on congenital penile curvature. Seven studies utilized penile models as their study subjects, while two studies involved patient participants. Every study had some component of a comparison method, like goniometer measurement, visual estimation, or comparison to the true penile model angle. Objective assessments of platform efficacy varied depending on the study, and included mean difference to comparison method, mean absolute error (MAE), and intraclass correlation coefficient (ICC). A summary of the typical workflow for the developed tools is illustrated in Fig. 2 and a summary of the extracted data from each article is included in Table 1.

**Table 1:** Summary of all identified studies

Automated At-Home Curvature Assessment in Peyronie’s Disease	
Author(s), Year(s)	Hsi et al. 2013 [12]; Brisbane et al. 2020 [13]
Objective	Develop and validate a smartphone application (UWPEN) for the at-home estimation of penile curvature
Study subjects	Penile models, Peyronie’s disease patients
Required input and model used	Two images and UWPEN application
Comparison	Goniometer measurement
Outcome	In ex-vivo testing of penile models, UWPEN curvature estimates closely aligned with goniometer measurement. In Peyronie’s patients, urologist estimation with UWPEN correlated well with goniometer estimates, however patient estimates using the application were likely less accurate.
Author(s), Year(s**)**	Witherspoon et al. 2022 [14]
Objective	Develop an AI tool for at-home curvature and plaque assessment
Study subjects	Peyronie’s disease patients
Required input and model used	Two images and a deep-learning model
Comparison	Physician estimate utilizing the same images
Outcome	Estimates utilizing their platform were similar to manual physician estimates utilizing the same images.
3D Imaging for Computational Assessment of Curvature	
Author(s), Year(s)	Walker et al. 2022 [15]
Objective	Create a platform for the objective estimation of penile curvature in Peyronie’s disease using 3D light-scanning.
Study subjects	Penile models
Required input and model used	3D light scanning and automated computational assessment
Comparison	Goniometer measurement and true model angle
Outcome	Estimates using 3D light-scanning technology followed by semi-automated estimation of curvature were more accurate than manual goniometer measurement
Author(s), Year(s)	Siapno et al. 2020 [16]
Objective	Improve accuracy and objectivity of angle estimation for hypospadias repair utilizing 3D light-scanning.
Study subjects	Penile models
Required input and model used	3D light scanning and automated computational assessment
Comparison	Goniometry measurement, 3D photogrammetry, traditional photogrammetry, and true model angle
Outcome	All three methods outputting 3D renderings were superior in accuracy and reliability when compared to goniometric measurement
Assessment of Congenital Penile Curvature	
Author(s), Year(s)	Fernandez et al. 2021 [17]
Objective	Develop a standardized and objective tool for the measurement of congenital penile curvature.
Study subjects	Penile models
Required input and model used	Singe image and semi-automated algorithm
Comparison	True model angle
Outcome	Their method demonstrated moderate accuracy in estimating penile curvature, with the average mean difference to true curvature ranging from 7.83° to 12.69°.
Author(s), Year(s)	Abbas et al. 2022, 2024 [18, 19]; Baray et al. 2023 [20]
Objective	Design and validate an AI platform for the objective assessment of congenital penile curvature.
Study subjects	Penile models
Required input and model used	Images and a deep learning model
Comparison	Visual inspection, goniometer inspection, mobile-phone-based application, and true model angle
Outcome	The AI-based platform was more accurate and reliable than all other methodologies evaluated.

## Discussion

### Automated At-Home Curvature Assessment in Peyronie’s Disease

In-office ICI followed by angle measurement utilizing either a protractor or goniometer is considered the gold standard for the assessment of penile curvature in Peyronie’s disease. However, this method is invasive, uncomfortable for patients, and some have argued that ICI does not accurately reflect a patient’s typical erection. In contrast, at-home estimation of penile curvature is known to be highly inaccurate, with patients often overestimating curvature by up to 20% [21]. Thus there is a need for objective and accurate tools to estimate penile curvature angle outside of the office. Hsi and colleagues (2013) sought to accomplish this by developing a smartphone application called the University of Washington Peyronie’s Examination Network (UWPEN) for accurate at-home penile curvature estimation [12]. The application requires the patient to capture an image of their erect penis from a superior and lateral view, which the application then converts into a black-and-white image with no anatomic detail. The patient then marks the vertex of the curvature and the two arms of the angle. The dorsal/ventral and lateral angulation is then automatically calculated by the smartphone application [12].

The application was first tested on ex-vivo malleable penile prostheses of variable curvature. The study evaluated the ICC between UWPEN, goniometer measurement and protractor measurement. The inter-test reliability between the three forms of measurement was ≥0.999 in all comparison groups, indicating UWPEN highly correlated with standard methods for angle measurement. Additionally, intra and interobserver reliability was high among urologists and non-urologists, indicating reproducible and consistent results in the hands of non-professionals.

Later on, Brisbane et al. (2020) published a follow-up study evaluating UWPEN in patients with Peyronie’s disease [13]. They recruited Peyronie’s disease patients with a clinical indication for pharmacologic erection (e.g. initial evaluation of curvature or evaluation of response to treatment). Following ICI, a urologist measured penile curvature with a goniometer followed by UWPEN, then the patient evaluated penile curvature utilizing UWPEN. The study found that urologist use of UWPEN for penile curvature assessment correlated well with the goniometer measurement (dorsal measurement: *R* = 0.87, lateral measurement: *R* = 0.77), while patient use of UWPEN did not correlate as closely to goniometer measurement (dorsal measurement: *R* = 0.55, lateral measurement: *R* = 0.62). In a survey, most patients indicated that their preferred method for measurement strategy was at-home use of UWPEN. While desirable by patients, the study demonstrated that at-home use of this smartphone application in its current form would likely not produce acceptable levels of accuracy for clinical decision-making. It is important to note the measurements recorded by the patients were their first attempt at using the application, and it may be that with additional practice correlation measurements may improve. Interestingly, the study also found that younger patient’s measurements tended to correlate more closely with the gold standard, suggesting possible technological barriers for older individuals.

In 2022, Witherspoon et al. developed a tool for at-home curvature assessment utilizing a residual neural network (ResNet), a deep learning model designed for image recognition [14]. The study was presented at the annual Sexual Medicine Society of North America conference, although it has not been published in a peer-reviewed journal. Images from the dorsal and lateral aspects of the penis in patients with Peyronie’s disease were taken, and ResNet was trained to identify the presence of penile curvature while removing background images. Linear lines were fit from the penile tip to the vertex of the curvature, then to the base of the penis, similar to the UWPEN tool. The accuracy of the algorithm’s measurement of penile curvature was compared to a trained urologist’s estimates utilizing the same images evaluated by the ResNet. The study found a median difference of 7.7 degrees between the model and the physician assessment. The study demonstrated promising initial data for the use of AI in penile curvature, although follow-up studies are needed to evaluate its effectiveness in the hands of patients for at-home curvature measurement, as well as comparisons to the current gold standards.

These studies created a promising foundation for future research and development into at-home software for penile curvature assessment. Continued research in this field has the potential to result in accurate, non-invasive measurement of penile curvature that improves overall patient comfort. However, several obstacles need to be overcome to achieve this. The developed tools need to be simple and have the flexibility to account for patient-related errors in their use. Additionally, as with all at-home penile curvature assessment, it can be more difficult to assess erection rigidity, which impacts curvature appearance. As such, with any tool developed for at-home use, there is an understanding that measured values may differ from physician-measured in-office estimates.

### 3D Imaging for Computational Assessment of Curvature

There is significant interobserver and intraobserver heterogeneity in the measurement of penile curvature, even in the hands of trained urologists utilizing the ICI followed by manual goniometer measurement [22]. With slight deviations in the estimation of the point of maximal curvature, curvature estimates can be altered to clinically significant degrees. This problem necessitates the development of accurate and reproducible platforms for penile curvature assessment. Walker et al. (2022) sought to accomplish this by utilizing novel 3D imaging for the computational assessment of curvature [15]. The researchers 3D-printed five models, two of which were angulated (40° cylinder and 68° realistic Peyronie’s penis). 3D structured light-scanning was then performed to re-digitize the models followed by computational estimation of curvature angle. Manual measurements utilizing a goniometer were performed by ten urologists, and both the computational and manual measurements were compared to the true values from the digital phantoms. There was no difference in angle estimates between the true standard value, urologist manual measurement, and light-scanned image for the 40° cylinder. However, in the realistic Peyronie’s penis model, the manual urologist measurement was significantly underestimated compared to the standard, while the 3D-light scanned model accurately estimated the angle of measurement to a near-perfect degree. The results of the study suggested that 3D imaging and computational assessment of curvature may be more accurate than manual angle estimation in Peyronie’s disease, although the findings have yet to be validated in human studies.

The use of 3D imaging for penile curvature assessment does not only apply to Peyronie’s disease, as Siapno et al. (2020) conducted a study to improve the accuracy and objectivity of angle estimation in hypospadias repair [16]. Utilizing six 3D printed blocks of various curvatures, they compared four methods for curvature assessment; manual with a goniometer, structured light scanning, traditional photogrammetry, and photogrammetry with a 3D camera. The latter three methods all outputted 3D renderings of the models followed by penile curvature assessment utilizing automated software. All three of the methods that utilized 3D renderings demonstrated high accuracy and precision, with each estimating curvature angle within 1 degree of the true value, and test-retest reliability was greater than 0.99 for each. In contrast, manual estimations with goniometers were often inaccurate by multiple degrees, and demonstrated moderate inter-rater reliability (ICC = 0.76). The study also provided information on the time needed for each method, with manual goniometry being the fastest (33 s), followed by light scanning and 3D camera photogrammetry (both < 10 min), then finally traditional photogrammetry (2 h). The study demonstrated that 3D renderings provide more accurate and precise measures of penile curvature when evaluating plastic models, although 3D imaging was more time-intensive and the results have not been validated in human studies. It is also important to consider how the added benefit of accuracy and precision is balanced with an increased cost associated with 3D imaging. Regardless, the initial research in this field is promising with regard to the accurate prediction of angle curvature.

#### Assessment of Congenital Penile Curvature

The field of pediatric urology in congenital penile curvature has pioneered many of the innovations in penile curvature evaluation. Penile curvature in a newborn is most frequently associated with hypospadias and requires surgical repair. Accurate estimation of the curvature angle in this population is of significant importance, as it directly relates to hypospadias severity and can guide surgical decision-making [23]. Currently, curvature angle is most often estimated with intra-operative goniometer measurement or visual assessment with artificial erection. However, both of these methods have been demonstrated to be inaccurate and unreliable [24, 25]. Newer and more accurate methods have been described, one of which was inspired by orthopedics surgery use of the Cobb angle, which is the angle generated by the joining of two perpendicular lines to the most-tilted vertebrae in scoliosis [26]. In addition, researchers in the field have been at the forefront of using AI and automated software to develop novel methods in curvature evaluation. This has important implications not only for hypospadias repair, but also for Peyronie’s disease. These technologies could likely be co-opted to estimate penile curvature in adults with Peyronie’s disease, expanding their usage beyond the cohort of patients with congenital penile curvature.

Fernandez et al. (2021) sought to standardize the measurement of congenital penile curvature utilizing a novel semi-automated algorithm [17]. Their method requires a single lateral image to be taken, followed by the manual identification of the tip and base of the penis. Then, a semi-automated algorithm identifies the geometric penile center and outputs an angle estimation. They tested their proposed algorithm on 9 penile models ranging from 10° to 90°, with images taken from 5 different camera angles (0°, 15°, 30°, 45°, 60°). They had 10 different evaluators estimate the angle of curvature utilizing their methodology at each camera angle. They found that the average mean difference between actual and estimated curvature ranged from 7.83° to 12.69°. In general, more accurate measurements were observed when utilizing a lower camera angle, with an optimal camera angle of 0°. The benefits of this methodology include its simplicity, requiring just a single image to be captured and minimal input from the user. However, as with other studies discussed, it has not been validated in human studies, thus its ability to evaluate more complex curvature deformities in real-world settings has yet to be seen.

Some of the most promising advancements regarding penile curvature assessment have come from Abbas et al. (2022, 2024) and Baray et al. (2023). The authors developed and validated a novel AI-based algorithm utilizing 2-dimensional images for the objective assessment of penile curvature. Their method can be split into three stages; penile area localization, shaft segmentation, and curvature estimation. First, the area where the penile model is located is identified, bounded, and cropped accordingly. Then, a deep learning architecture called U-net is used to generate binary masks that discriminate the penile shaft from background. Finally, the segmented shaft is evaluated using a custom angle estimation algorithm which outputs a curvature estimation. In the first publication describing their method, 9 models were 3D printed with uniplanar radial curvatures ranging from 18° to 88° that realistically resembled a typical penile curvature [18]. Each stage of their method was independently assessed on these models, and they found a high degree of precision in penile area localization and accurate penile shaft segmentation. Compared to the true angle of curvature for each model, most estimates were within 5 degrees, and the MAE across all models was 8.53. To improve the accuracy of angle estimation, they performed a follow-up study with alterations to their AI-based approach [20]. With this new approach, they utilized HRNet, a deep learning architecture, to identify four key points along the penile shaft. They utilized a 4-point approach (in contrast to the traditional 3-point approach) to account for both hinge-type and arc-type shafts. The new approach led to a remarkable improvement in prediction accuracy on the same models previously tested, with an observed MAE of just 3.81, more than twice as accurate as the previous method. Additionally, the new approach was tested on four patient cases, utilizing lateral images of ventral congenital penile curvature. The measured angles were similar to that of a physician’s estimate utilizing a mobile application.

After developing this AI-based approach to curvature estimation, they then moved to validate the study in the hands of thirty-five clinicians [19]. The participants measured seven 3D-printed models ranging from 33° to 88° utilizing four different methods; visual inspection, goniometer measurement, mobile-phone-based application, and their previously developed AI algorithm. The study found that across all 4 measurement methods, the AI-based framework was the most accurate method, with an MAE of just 9.07. In contrast, visual inspection was the least accurate method and had an MAE of 15.56. Similarly, the visual inspection method had the lowest inter-rater ICC at 0.546, while the AI-based framework had the highest at 0.753. Visual inspection had the benefit of being the fastest of all methods, averaging just 8 s, although the AI-based approach was itself quite fast averaging 22 s. In summary, the AI framework for penile curvature assessment developed by Abbas et al. is superior in accuracy and reliability to alternative methods of visual inspection and goniometer measurement in this controlled setting. One limitation of the AI-based tool was lesser performance on low or intermediate curvature angles compared to larger angles. Despite this, even at less severe angles the AI tool generally performed better than other methods. The study’s strengths include its realistic modeling and testing on practitioners unfamiliar with the technology. While these features could lend themselves to an easy transition into the clinic setting, the technology needs to be validated in human studies.

## Conclusion

Over the past decade, considerable advancements have been made in the assessment of penile curvature for both Peyronie’s disease and congenital penile curvature. Many of these advancements have been driven by AI and computational platforms that allow for the reproducible, objective, and accurate estimation of angle curvature. Research in this field is still in its beginning stages, and further studies are needed to evaluate how these platforms perform in clinical environments. While these emerging technologies show significant promise, it is important to consider the ethical and appropriate use of AI in medical settings. In most scenarios, AI in healthcare will function as *augmented intelligence*, meaning AI technology will perform a supportive role to clinicians in the care of patients [27]. The researchers in the studies discussed have demonstrated how AI can effectively complement human expertise, underscoring the importance of maintaining a supportive role for AI in healthcare settings. Given the rapid pace of research and development in this field, we can anticipate significant changes in how penile curvature is evaluated in the coming years.

## Key References

- Brisbane WG, Rogers MJ, Hsi RS, Rajanahally S, Schade GR, Trew L, et al. Comparison of clinician and patient users of a mobile phone application to assess penile curvature in Peyronie’s disease. Int J Impot Res. 2020 Jul;32(4):401–8
  - Well designed study at the forefront of at-home curvature assessment in Peyronie's disease.
- Siapno AED, Yi BC, Daniels D, Bolagani A, Kwan L, Walker D, et al. Measurement accuracy of 3-Dimensional mapping technologies versus standard goniometry for angle assessment. J Pediatr Urol. 2020 Oct;16(5):547–54
  - Demonstrated the superior accuracy of 3D technology for curvature assessment compared to traditional techniques.
- Abbas TO, AbdelMoniem M, Villanueva C, Al Hamidi Y, Elkadhi A, AlSalihi M, et al. Urologist validation of an artificial intelligence-based tool for automated estimation of penile curvature. J Pediatr Urol. 2024 Feb;20(1):90.e1-90.e6
  - Groundbreaking study demonstrating the promise of AI in penile curvature assessment.

## Supplementary Information

Below is the link to the electronic supplementary material.

Supplementary file1 (DOCX 86.2 KB)

## Data Availability

No datasets were generated or analysed during the current study.

## Abbreviations

AI: Artificial Intelligence
3D: Three-Dimensional
ICI: Intracavernosal injection
MAE: Mean Absolute Error
ICC: Intraclass Correlation Coefficient
UWPEN: University of Washington Peyronie’s Examination Network
